# H3K36me3, message from chromatin to DNA damage repair

**DOI:** 10.1186/s13578-020-0374-z

**Published:** 2020-01-31

**Authors:** Zhongxing Sun, Yanjun Zhang, Junqi Jia, Yuan Fang, Yin Tang, Hongfei Wu, Dong Fang

**Affiliations:** Life Sciences Institute, Zhejiang University, Hangzhou, 310058 Zhejiang China

**Keywords:** H3K36me3, DNA damage, Oncohistones, Homologous recombination, Nonhomologous end-joining, DNA mismatch repair, Clear cell renal cell carcinoma, Acute myeloid leukemia, Diffuse intrinsic pontine glioma

## Abstract

Histone marks control many cellular processes including DNA damage repair. This review will focus primarily on the active histone mark H3K36me3 in the regulation of DNA damage repair and the maintenance of genomic stability after DNA damage. There are diverse clues showing H3K36me3 participates in DNA damage response by directly recruiting DNA repair machinery to set the chromatin at a “ready” status, leading to a quick response upon damage. Reduced H3K36me3 is associated with low DNA repair efficiency. This review will also place a main emphasis on the H3K36me3-mediated DNA damage repair in the tumorigenesis of the newly found oncohistone mutant tumors. Gaining an understanding of different aspects of H3K36me3 in DNA damage repair, especially in cancers, would share the knowledge of chromatin and DNA repair to serve to the drug discovery and patient care.

## Background

In eukaryotes, the genomic DNA is packaged into chromatin to maintain the higher order structure. Nucleosome, the smallest subunit of chromatin, consists of 146–147 base pairs of DNAs wrapped around an octamer of core histone proteins, including one H2A–H2B tetramer and two H3–H4 dimers [1]. The N- and C-terminal tails of core histones are enriched with basic amino acids and may undergo post-translational modifications during distinct cellular processes, such as gene transcription, cell cycle checkpoint, centromere assembly, heterochromatin formation, DNA replication and DNA repair [2–5]. Different modifications have been reported, at least by Mass Spectrometry analysis, on core histones, of which the mostly studied modifications are methylation, acetylation, phosphorylation, ubiquitylation and SUMOylation [6].

Histone methylations usually occur on the arginine or lysine residues. Histone H3 is methylated at lysine 36 (H3K36) with mono-, di- and tri-methylations (H3K36me1/me2/me3). In yeast, Set2 is the solo enzyme responsible for all of these three forms of methylations [7]. In mammalian cells, several redundant enzymes, including NSD1 [8], NSD2 [9], NSD3 [9, 10], ASH1L [11], SETD3 [12], SETMAR [13], and SMYD2 [14], are able to mono- and di-methylate H3K36. SETD2, the paralogous protein of Set2, is the only enzyme found to catalyze the formation of H3K36me3, while there are still arguments that it also methylates H3K36 to H3K36me1 and H3K36me2 in vivo [15]. Several lines of evidences have shown that H3K36me3 plays a role in the transcriptional activation. H3K36me3 is tightly correlated with actively transcribed genome regions [16, 17]. SETD2, the methyltransferase for H3K36me3, is recruited through the Ser2 phosphorylated C-terminal domain (CTD) of RNA polymerase II (RNAPII) during gene transcription elongation, while the Ser5 phosphorylation of RNAPII is the characteristic of the paused polymerases at promoters [18]. Genome-wide studies show that H3K36me3 distributes in the gene body in a 3′ end enriched manner like the Ser2 phosphorylated RNAPII [19, 20]. In addition, H3K36me3 acts as a safeguard to prevent aberrant transcriptional initiation from cryptic gene promoters [21, 22]. In yeast, Set2 secures H3K36me3 co-transcriptionally and recruits the reduced potassium dependency 3 small (Rpd3S) through its chromodomain-containing subunit ESA1 associated factor 3 (Eaf3), which will then subsequently deacetylate histones around the transcribed gene body regions [23]. Through this process, cells maintain the deacetylated chromatin to inhibit the cryptic transcription. In mammalian cells, SETD2 is recruited to the RNAPII elongation complex through an Spt6:Iws axis [24]. However, depletion of SETD2 does not affect the histone acetylation across the gene coding regions, indicating that the H3K36me3 preserves the repressive chromatin status independent of histone acetylation. Another important role of H3K36me3 in gene expression is to regulate RNA splicing [25]. To regulate the RNA splicing machinery, H3K36me3 forms an adapter system with MORF-related gene 15 (MRG15) to recruit splicing regulator polypyrimidine tract–binding protein (PTB) [26]. Deletion of 3′ splice site of genes causes a shift of H3K36me3 from 5′ ends to 3′ ends, despite the fact that mutations of poly(A) site have no apparent effects on H3K36me3. Moreover, a global inhibition of splicing also triggers the repositioning of H3K36me3 [27]. These results suggest that H3K36me3 and co-transcriptional splicing complex interact with each other. Since H3K36me3 is tightly associated with gene expression and RNA splicing, how does H3K36me3 participate in other cellular processes like DNA damage repair?

## Main text

### Maintain genome integrity after DNA damage

Cells are constantly facing DNA damaging agents from both endogenous and exogenous origins [28]. These bulky DNA lesions need to be repaired by naturally adapted DNA repair machineries [29]. If unrepaired or misrepaired, DNA lesions may cause the accumulation of DNA errors and great threat to genome stability, which is a hallmark of cancer, aging, neurodegeneration, and immune deficiencies [30]. DNA double strand breaks (DSBs) usually arise by the attack of electrophilic molecules like reactive oxygen species, leading to lesions in both DNA strands of the double helix [31]. DSBs are majorly perceived by ataxia telangiectasia mutated (ATM) kinase which is critical for the immediate DNA damage response (DDR) [32]. ATM phosphorylates and regulates the activity of several substrates in DNA repair, including p53-binding protein 1 (53BP1). To maintain genome stability, cells choose from two different pathways to repair DSBs: homologous recombination (HR) and nonhomologous end-joining (NHEJ) [33]. In the HR pathway, helicases and nucleases are recruited to resect 5′ DNA ends to generate two 3′ single-stranded DNA overhangs. The 5′ end resection is done by removing a short oligonucleotide through the activities of C-terminal binding protein interacting protein (CtIP) and the Mre11-Rad50-Nbs1 (MRN) complex, followed by Exo1 or DNA2-BLM [34]. Phosphorylated replication protein A (RPA) binds to the 3′ single-stranded DNA overhangs and removes DNA secondary structures which, in turn, leads to the RAD51 nucleofilament formation. The RAD51 filament will then promote strand invasion of a homologous DNA and subsequently the accurate repair of DSBs [34, 35]. Cells can also repair DSBs through NHEJ. During classical NHEJ, the broken DNA ends are rapidly bound and blocked by Ku70–Ku80 heterodimer (Ku) which protects the DNA from 5′ end resection and holds the broken DNAs in a close proximity [36]. DSBs are then processed and joined by the Ligase 4 (Lig4), XRCC4, XLF complex [36]. DSBs can also be repaired by alternative NHEJ pathways, such as microhomology-mediated end-joining (MMEJ), without the recruitment of Ku or Lig4. MMEJ is initiated through end resection like HR, but followed by the end-joining through short direct repeats of microhomology [37]. In contrast to HR, which recruits homologous sequences outside the DNA replication process to promote an error-free DNA repair, NHEJ directs ligations of the DSB ends in an error-prone manner throughout the cell cycle. Both repair pathways depend on the DNA damage sensors, transducers, and effectors to detect and repair the breaks. Since all these evens happen on the chromatin, the diversity of histone modifications on chromatin may affect the choice between HR and NHEJ.

### H3K36me3 in the error-free repair (HR)

The early clues of the participation of H3K36me3 in DNA repair are from small scale genetic screening. In *Saccharomyces cerevisiae*, overexpression of RPH1 which demethylates H3K36me3 primarily leads to a growth defect in response to UV irradiation [38]. In addition, the H3K36me3 catalyzing enzyme, Set2, is involved in hydroxyurea (HU)-induced replication checkpoint activation [39]. Besides these signs of H3K36me3 in DNA repair, emerging evidences show that Pro-Trp-Trp-Pro (PWWP)-domain containing proteins recognize H3 lysine methylation and act as anchors between histone methylation and downstream effectors, including 53BP1 [40, 41]. The early molecular mechanisms of how H3K36me3 participates in the DSBs were reported in 2012. Psip1 encodes two protein isoforms by alternative splicing, p52 and p75 [42]. The p75 isoform, also known as lens epithelium derived growth factor (LEDGF), is a chromatin-associated protein involved in cancer, auto immune diseases, HIV pathogenesis, and cell survival. LEDGF/p75 facilitates HR in S- and G2-phase cells and depletion of p75 sensitizes cells to ionizing radiation, camptothecin and mitomycin C [43]. LEDGF/p75 binds CtIP in a DNA damage-dependent manner, therefore enforcing the access to DSBs to promote DNA repair. Moreover, the N-terminal PWWP domain of p75 specifically recognize H3K36me3. This binding is critical for the constitutive association of p75 with chromatin. A PWWP-mutated (W21A) p75 which does not bind histones or chromatin compromises the camptothecin-induced association of p75 and CtIP. The ability of p75 to protect cells against camptothecin-induced cytotoxicity is also diminished by the W21A mutation. The finding of p75 associated with H3K36me3 in DNA repair highlights the direct role of H3K36me3 in the regulation of HR. In the report by Pradeepa et al*.* [44], the p52 isoform, lacking the C-terminal of long isoform p75, was shown to recognize H3K36me3 by the N-terminal PWWP domain and associate with active transcribed genes. When cell lysates are immunoprecipitated by p52, the mass spectrometry reveals that around 95% of p52 binding proteins are known to function in pre-mRNA processing whereas Srsf1 is one of the major hits [44]. Depletion of p52 by gene trap in mouse embryonic stem cells leads to a reduced level of H3K36me3-associated Srsf1. Through microarray and RT-PCR analysis, the p52 loss is found to cause an alternative RNA splicing. More importantly, the distribution of Srsf1 is mispresented around the alternatively spliced exons with p52 loss. Together, despite of a similar N-terminal PWWP domain like p75, p52 plays a critical role in modulating mRNA splicing [44]. In this study by Pradeepa et al*.*, the function of p52 in DNA damage repair pathway is not tested because the depletion systems used are gene trap or knock out of Psip1 which deplete both p52 and p75. Whether depletion of p52 affects the HR pathway or complementation of p52 in the Psip1 knockout system would rescue the HR pathway needs to be further analyzed.

To explore the DSBs in cells, researchers developed an artificial system to introduce DSBs in genome: stable cell lines expressing a restriction enzyme (AsiSI) fused to a modified estrogen receptor (ER) ligand-binding domain, which is directed to nucleus under the control of 4-hydroxytamoxifen (4OHT). Nuclear localization of the AsiSI could rapidly generate around 150 DSBs across the genome [45]. When DSBs are introduced in cells by this system, RAD51, which promotes homologous strand invasion during HR, is recruited to the chromatin by H3K36me3 at actively transcribed genes [46]. Depletion of either component of H3K36me3:p75 axis disrupts the RAD51 binding. Moreover, this recruitment of RAD51 is also H3K36me3-dependent in laser-induced DNA damage and I-SceI–induced DSBs. Interestingly, H3K36me3 is not induced at any DSBs sites under AsiSI activation, indicating that H3K36me3 pre-exists at the actively transcribed genes to get these genomic loci ready for the repair of DSBs [46–48]. Besides RAD51, the H3K36me3:p75 axis also facilitates the RPA binding to DNA damage sites to promote HR [49]. Reducing H3K36me3 levels by overexpressing of H3K36 demethylase KDM4A diminishes HR repair events [49]. In addition, H3K36me3 cross-talks with other histone marks, like H4K16ac, by facilitating the interactions of corresponding histone acetyl transferase with DNA repair complex [50]. In addition to p75, MRG15, a histone binding protein, recognizes H3K36me3. MRG15 plays as an adaptor to load PALB2, which is critical for the strand invasion during HR [51]. Like p75, the recruitment of PALB2, occurring before the DSBs, ensures an immediate response to DNA stress [51]. Besides these downstream effectors, H3K36me3 is also important for the activation of early sensors in DSBs. Although the detail mechanism is not clear, ATM activation, which is the direct sensor in early DNA damage signaling, is impaired by the depletion of SETD2 [48]. One interesting observation is that PRDM9, which is a meiosis-specific histone methyltransferase responsible for H3K4me3 and H3K36me3, mediates the DSB formation through its methyltransferase activity in testis [52]. PRDM9-specified H3K4me3 sites are analyzed for the competition of DSB formation. The PRDM9-mediated H3K36me3 sites may also participate in the DSB formation indicating a dynamic of H3K36me3 upon DSB formation.

In fission yeast, Set2-dependent H3K36 methylation and Gcn5-dependent H3K36 acetylation antagonistically control the selection of HR and NHEJ pathway [53]. H3K36 methylation reduces chromatin accessibility and promotes NHEJ, while H3K36 acetylation increases chromatin accessibility and induces HR. Depletion of either Set2 or Gcn5 primes DNA repair to the other pathway. In fission yeast, H3K36me3 levels increase to a peak in G1 phase when NHEJ prevails, while H3K36me2 and H3K36ac increases after G1 release when HR predominates. Moreover, base excision repair (BER) of alkylation damage induced by methyl methanesulfonate is regulated by H3K36me3 in budding yeast. High levels of pre-existing H3K36me3 are coupled with lower BER at distal translation locations and paradoxically more rapid repair at translational positions near the nucleosome dyad [54]. Set2 is the only enzyme responsible for H3K36me1/me2/me3 in yeast, which makes it challenging to distinguish the functional differences between H3K36me2 and H3K36me3 in DNA damage repair. In consistent with the distinct functions of H3K36me2 and H3K36m3, SETMAR-mediated H3K36me2 is generated after ionizing radiation and recruits NBS1 and Ku to promote NHEJ in the response of DSBs in human cells [55].

### H3K36me3 in the “quick and dirty” repair (NHEJ)

Interestingly, SETD2 promotes both 53BP1 and RAD51 recruitments to DSBs during DNA repair [48]. Chromatic binding of 53BP1 at DSBs promotes NHEJ by inhibiting 5′ end resection of the DNA breaks, while the recruitment of RAD51 tiles the repair pathway to HR. This contradictory recruitments of 53BP1 and RAD51 raise the possibility that H3K36me3 functions in the regulation of NHEJ. Besides the PWWP domains, Tudor domains are known to recognize methylated histone proteins [56]. In human cells, the PHD finger Protein 1 (PHF1) which contains Tudor domain is associated with DNA damage repair proteins. PHF1 is recruited to DSB sites depending on Ku that is the sensor for NHEJ. Moreover, depletion of PHF1 sensitizes cells to X-ray induced DNA damage and increases HR frequency [39]. The mechanisms of how PHF1 functions in DDR are reported in the structure studies [57]. The Tudor domain of PHF1 preferentially binds to H3K36me3 peptides among H3K4, H3K9, H3K36 methylated and unmodified peptides. A 1.9 Å X-ray crystallographic structure shows that the side chain of H3K36me3 peptide interacts with W41, Y47, F65 and F71 residues of PHF1. The recognition between H3K36me3 and PHF1 inhibits H3K27me3 in vitro and in vivo. In the sense that H3K27me3 is the mark for silenced genomic loci, these findings suggest that the retention of PHF1 at DNA damage sites maintains an open chromatin. Further studies show that the Tudor domain interacts with nucleosome core particle (NCP) containing a methyl-lysine analogue at position 36 of histone H3 (H3K_C_36me3-NCP). This interaction depends not only on the tri-methylation of H3K36 but also on the DNA site within the NCP [58]. Through this parallel interaction, PHF1 facilitates in vitro association of the transcription factor LexA to the DNAs wrapped around nucleosomes, which is inaccessible in the fully wrapped nucleosome. These findings suggest a role of PHF1 to read H3K36me3 to support an open chromatin for the efficient DNA repair. Moreover, PHRF1, being associated with RNAPII upon DNA damage [59], modulates NHEJ through the interactions with H3K36me2 and H3K36me3 [60].

H3K36me3 regulates both HR and NHEJ pathways, where LEDGF/p75 and MRG15 are the major readers for HR and PHF1 is the major reader for NHEJ in mammalian cells. Since these readers are all expressed in cells, the choice of repair pathway may depend on which readers are abundantly present on the H3K36me3 marked chromatin. The cell systems used for cellular phenotype analysis are HEK293T cells in the study of PHF1 [57] whereas U2OS, Hela and MEF cells are used to detect the association of LEDGF/p75 and MRG15 with H3K36me3 in HR pathway [43, 51]. It will be interesting to test whether the protein levels of readers are different or other adapters are participated in the recruitments of readers. It is also possible that other histone marks may participate in the decision of DSB repair pathway choice [61]. For instance, H3K36me2, H4K20me1/me2, H3K79me2 and H4K16ac are involved in the DSB repair to promote NHEJ [55, 62–65]. Moreover, these histone marks are mainly correlated with active gene transcription like H3K36me3. Comprehensive analysis of H3K36me3 and other co-exist histone marks at DSB sites may help to dissect the combined histone marks in DSB repair pathway choice.

### H3K36me3 in the repair of small lesions (DNA mismatch repair)

Besides the DSBs, base–base and small insertion/deletion mispairs which are generated during DNA replication are repaired by DNA mismatch repair (MMR). The sensors for MMR in human cells are hMSH2-hMSH6 (hMutSα) and hMSH2-hMSH3 (hMutSβ) [66]. Defects in MMR cause microsatellite instability (MSI) as a hallmark of cellular phenotype [67]. Reconstituted nucleosomes with mismatch-containing DNA are poor substrate for the in vitro MMR system, indicating that histone modifications and reader proteins are needed in MMR [68]. The hMutSα is recruited onto chromatin through the PWWP domain of hMSH6 which directly interacts with H3K36me3. Interestingly, the abundance of hMutSα foci correlates with the levels of H3K36me3 throughout the cell cycle, where their levels reach to highest in the early S phase, to modest in the middle S phase, and to the lowest in late S and G2/M phases. As in HR repair, H3K36me3 preloads to the genomic loci to recruit MMR machinery hMutSα before mispairs are introduced during DNA replication [69, 70]. More importantly, MMR-competent nuclear extracts could not repair the mismatch located between two nucleosomes with H3K36me3, further supporting the idea that H3K36me3 pre-sets the genomic loci with hMutSα, once other MMR signals and DNA replication are sensed, to trigger the DNA repair. Genome-wide studies show that H3K36me3-mediated MMR is preferentially enriched at exons and actively transcribed regions to safeguard transcribed genes [71]. Phenotypically, cells with H3K36me3 deficiency exhibit increased frequency of spontaneous mutations and MSI. Overexpression of KDM4A-C which catalyze the demethylation of H3K9me2/3 and H3K36me2/3, but not KDM4D which demethylates only H3K9me2/3 interrupts hMutSα foci formation during S phase. Moreover, cells overexpressing KDM4A-C show reduced MMR [72]. These observations demonstrate that H3K36me3 also functions in the MMR to maintain genome stability.

### H3K36me3 associated DDR in tumorigenesis

H3K36me3 may negatively impede DSBs by restricting chromatin accessibility through nucleosome positioning or, more directly, by favoring the repair of DSBs [73, 74]. Disruption of H3K36me3 coupled DSBs repair inhibits the immunoglobulin V(D)J rearrangement in B cells [75] and promotes tumorigenesis of aggressive cancers, such as clear cell renal cell carcinoma (ccRCC) [76], acute myeloid leukemia (AML) [77], and diffuse intrinsic pontine glioma (DIPG) [78].

SETD2 mutations are identified in ccRCC cells as homozygous truncating mutations and copy number loss [79–81]. Besides reduced DNA damage repair by HR, SETD2 mutant ccRCC tumors show altered chromatin organization occurred primarily at actively transcribed genes, leading to intron retention and aberrant splicing [47]. Decrease of H3K36me3 results in alternative exon usage in RNA splicing in ccRCC tumors [82]. Moreover, the genes exhibiting aberrant splicing, including TP53, ATR, PTEN, and CCNB1, are tumor suppressors whose inactivation will promote tumorigenesis [80]. SETD2-mutant ccRCC cells fail to activate p53 by phosphorylation and increased protein level which is critical as a master guardian of the genome after DNA damage, showing disturbed cell-cycle checkpoint activation and reduced cell survival [76]. These findings elucidate the observation that, despite a low frequency of TP53 mutation, the p53 cell-cycle checkpoint is usually defected in ccRCC tumors. In addition, SETD2 depletion in ccRCC cells reveals aberrant nucleosome compaction and chromatin association of the key replication protein MCM7 and DNA polymerase delta [83, 84].

AML cells usually bear SETD2 mutations which abolish the preload DDR components. The impaired DDR inhibits cellular apoptosis induced by DNA damaging agents and increases spontaneous mutations at sites of reduced H3K36me3 [77, 85]. Treatment of KDM4A (H3K9 and H3K36 demethylase) inhibitors restores H3K36me3 levels and sensitizes AML cells to DNA damaging agents. Besides the role in DNA damage repair, SETD2 is also required to maintain high H3K79me2 levels in AML cells. Pharmacologic inhibition of DOT1L, the H3K79 methyltransferase, synergizes with SETD2 mutations to induce DNA damage, growth arrest, and apoptosis, indicating a cross-talk of H3K36me3 and H3K79me2 in AML cells [86, 87].

MMR-deficient tumors selectively acquire single-nucleotide variants (SNV) in regions with active histone marks, especially H3K36me3, indicating a role of H3K36me3-dependent MMR in tumorigenesis [88]. Recently, with the expansion of large-scale genomic sequencing, somatic mutations of the histone proteins are found in a variety of tumors. These histone mutations play a role like oncogenes to promote tumorigenesis, so that the high frequent histone mutations are also called oncohistones [89]. An oncohistone H3 mutation is detected at a high frequency in aggressive DIPG cases, leading to a glycine 34 to arginine or valine mutation (H3G34R/V) [90, 91]. Biochemistry studies have shown that these H3G34 mutations, which substitute non-side chain residues with large side chain residues, reduce the corresponding H3K36me3 on the H3G34R/V histone proteins particularly through the inhibition of H3K36 methyltransferase SETD2 [78]. More importantly, this inhibition of H3K36me3 occurs at the incorporation sites of H3G34 mutations [92]. In human cells, there are 15 copies of genes encoding canonical histone H3.1/H3.2 and histone H3.3 variants while H3K36 is conserved in all histone H3 proteins. How does this single heterozygous mutation in 15 genes lead to tumorigenesis? In fission yeast that expresses only mutant H3G34R, DNA damage repair by HR is diminished and the DNA repair dynamics at the compromised replication fork are delayed [93]. However, it’s known that H3K36me3 controls NHEJ in fission yeast [53]. The H3G34R mutation may lead to the observed defects of HR in an indirect way. Although this model does not mimic the in-situ status of H3G34R mutation in DIPG cases because these yeast cells are modified to express only the mutant H3, it sheds light on the functions of reduced DNA damage repair and increased genome instability in the tumorigenesis of DIPG. In human cells, H3G34R/V mutations block the interactions of H3K36me3 and hMutSα, preventing the loading of MMR complex to chromatin [78]. Cells bearing H3G34R/V mutations display a week mutator phenotype, such as increased MSI and higher drug-induced mutation rate. Therefore, H3G34 mutations promote genome instability and possibly tumorigenesis by impeding MMR activity. Besides these known results, one very interesting hypothesis is that whether the G34R mutation itself would generate a new site for methylation on the histone tail [94].

H3G34R/W/L mutations are also found in the giant cell tumor of bone, indicating a DNA damage repair deficiency in these tumors [95]. In addition to the H3G34 mutations, H3K36M mutations, found in over 90% of chondroblastoma cases and in large subgroup of head and neck sarcomas, function as dominant negative regulators to reduce H3K36me2 and H3K36me3 on wild type histone H3 [95–98]. H3K36M oncohistones reduces H3K36me2 and H3K36me3 genome-wide through the inhibition of at least two histone H3K36 methyltransferases, NSD2 and SETD2 [96–99]. Knock-in of heterozygous H3.3K36M mutation into chondrocytes reduces the H3K36 methylation and subsequent expression of cancer related genes. Moreover, the changes of gene expression and H3K36 methylation are highly correlated [96]. In mouse mesenchymal progenitor cells, the heterozygous H3.3K36M mutation causes the global decrease of H3K36 methylation as well as defects of cell differentiation [97]. H3K36 methylation is known to be antagonistic to H3K27me3. Indeed, H3K27me3 increases in the intergenic regions where the H3K36me2 is decreased in the H3.3K36M mutate cells [97, 98]. How this H3K36M mutation controls DNA damage repair is still unknown [94, 99]. More efforts should be placed to discover the detailed molecular mechanisms of how oncohistones promote tumorigenesis through DNA damage repair pathways.

### Treatment of tumors with diminished H3K36me3

**Fig. 1:**
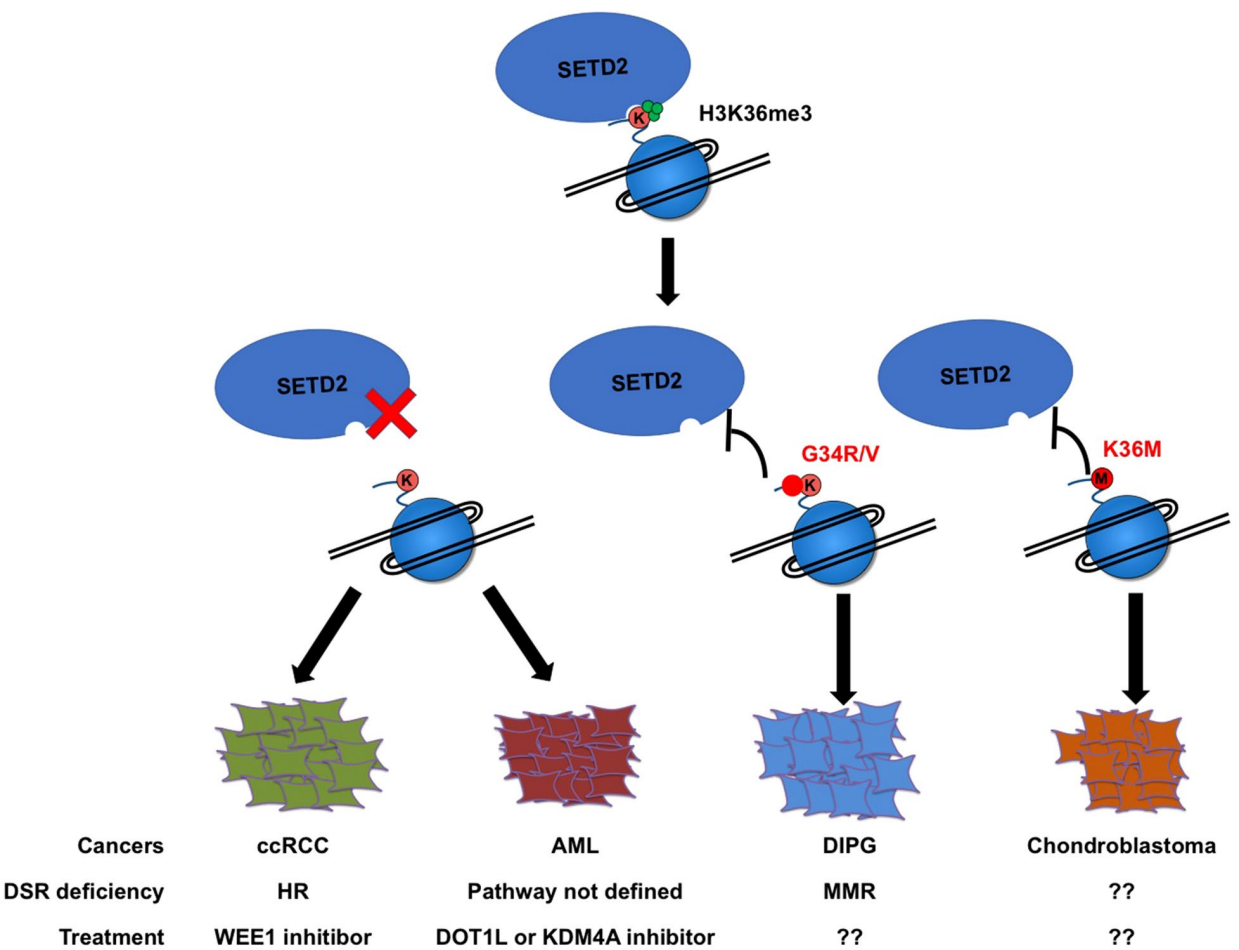
Reduction of H3K36me3 leads to tumorigenesis. Cells with the SETD2 mutation show reduced H3K36me3 and DSR deficiency. H3G34R/V mutation and H3K36M mutation can inhibit the enzymatic activity of SETD2 to reduce the H3K36me3 in cells

One well known inhibitor for H3K36me3 deficient ccRCC is the WEE1 inhibitor AZD1775, which acts synthetically with H3K36me3 depletion to reduce the tumor cell growth. AZD1775 and H3K36me3 both target RPM2 which is the ribonucleotide reductase subunit responsible for dNTP uptake. Decreased H3K36me3 represses the gene expression of RPM2, whereas WEE1 inhibition degrades the RPM2 protein by CDK activation. Thus, H3K36me3 depletion and WEE1 inhibition regress cancer cell growth by dNTP starvation [100]. More importantly, this synthetic lethality is unlikely dependent on HR deficiency [100]. However, the WEE1 inhibitor is not suitable for the treatment of AML cells. In AML model with the SETD2 mutations, RPM2 protein levels are unaffected, indicating the cell-context dependent effects of SETD2 loss on gene expression. Because SETD2 binds to and regulates different subsets of genes in distinct cells, depletion of SETD2 causes direct and indirect gene expression changes which may also affect specific cellular processes including DNA damage repair [77, 85]. In consistent with this hypnosis, loss of Msh2 and a single-radiation hit in mice induce H3K36me3 alternations at the originally H3K4me3 marked genes, which are enriched in DNA repair, RNA processing, and ribosome biogenesis [101]. There are still no druggable targets found in other tumors, like DIPGs bearing H3G34R/V mutation and chondroblastomas with H3K36M mutation (Fig. 1). Since H3K36me3 deficient cells have reduced abilities of HR and MMR, or maybe NHEJ, it would be interesting to test whether blocking of other DNA repair pathways will act like a synthetic lethal interaction to sensitize these cancer cells to DNA damaging agents. Other screens, such as large-scale inhibitor screens and genetic screens, may lead to further understandings of how these aggressive SETD2 mutant cells can be targeted.

## Conclusions

Chromatin structure dynamics, which represent the alternations between tight and loose nucleosome regions, may affect the accessibility of DNA damage repair machinery on the damaged sites and subsequently the repair efficiency. H3K36me3 serves as the postman to send chromatin information to DNA damage repair processors. In HR, NHEJ and MMR, H3K36me3 functions as the linker to pre-set the DSR complex at actively transcribed genes. This “set and go” system ensures the quick response to DNA damage. Emerging result from the PRDM9 also indicates the dynamic deposition of H3K36me3 at DNA damage sites in testis. In the sense that H3K36me3 can also regulate the gene expression and RNA splicing, H3K36me3 may participate in the DSR by disturbing the gene expressions of DNA repair proteins. So, how to distinguish the double faces of H3K36me3 in DSR may be an interesting filed to explore. More importantly, evidences show that H3K36me3 directly regulates HR, NHEJ and MMR repair machinery in cells. How the reduction of H3K36me3 affects only one DNA repair pathway in specific cells is still not clear. Because of the scopes of this review, the functions of other H3K36 methylations, especially H3K36me2 which is induced upon DNA damage, are not discussed. Whether and how, if so, the H3K36me2 and H3K36me3 cross-talk to regulate the DSR is also warranted.

## Data Availability

Not applicable.
